# An ultra scale‐down methodology to characterize aspects of the response of human cells to processing by membrane separation operations

**DOI:** 10.1002/bit.26257

**Published:** 2017-02-23

**Authors:** Maria Fernanda Masri, Kate Lawrence, Ivan Wall, Michael Hoare

**Affiliations:** ^1^ The Advanced Centre for Biochemical Engineering, Department of Biochemical EngineeringUniversity College LondonGordon StLondonWC1H 0AHUK; ^2^ Centre for Commercialization of Regenerative MedicineThe Banting Institute Suite110‐100 College StreetTorontoOntarioCanada; ^3^ ImmunocoreAbingdonOxonUK

**Keywords:** ultra scale‐down, cell membrane integrity, cell therapy, lactate dehydrogenase, human cells, membrane separation

## Abstract

Tools that allow cost‐effective screening of the susceptibility of cell lines to operating conditions which may apply during full scale processing are central to the rapid development of robust processes for cell‐based therapies. In this paper, an ultra scale‐down (USD) device has been developed for the characterization of the response of a human cell line to membrane‐based processing, using just a small quantity of cells that is often all that is available at the early discovery stage. The cell line used to develop the measurements was a clinically relevant human fibroblast cell line. The impact was evaluated by cell damage on completion of membrane processing as assessed by trypan blue exclusion and release of intracellular lactate dehydrogenase (LDH). Similar insight was gained from both methods and this allowed the extension of the use of the LDH measurements to examine cell damage as it occurs during processing by a combination of LDH appearance in the permeate and mass balancing of the overall operation. Transmission of LDH was investigated with time of operation and for the two disc speeds investigated (6,000 and 10,000 rpm or *ϵ*_max_ ≈ 1.9 and 13.5 W mL^−1^, respectively). As expected, increased energy dissipation rate led to increased transmission as well as significant increases in rate and extent of cell damage. The method developed can be used to test the impact of varying operating conditions and cell lines on cell damage and morphological changes. Biotechnol. Bioeng. 2017;114: 1241–1251. © 2017 The Authors. Biotechnology and Bioengineering Published by Wiley Periodicals, Inc.

## Introduction

The cell‐based therapy industry is rapidly growing and becoming increasingly important, having led to the emergence of several new products approved for clinical use and many more currently in clinical trials (Coopman and Medcalf, 2014). In the past two decades, the industry has seen the approval and commercialization of the first cell‐based therapies (Brandenberger et al., 2011). Yet, a number of challenges remain in getting new therapies to market. The production and subsequent bioprocessing of the cells is of utmost importance, as the product and process have become inseparable (Mason and Hoare, 2006). This is primarily because the end‐products are defined by their respective manufacturing process.

The exposure to a range of mechanical and physicochemical stresses during processing can cause internal adjustments in the cell. These adjustments can lead to physiological and metabolic changes (Acosta‐Martinez et al., 2010; Al‐Rubeai et al., 1995b; Zoro et al., 2008), complete lysis or programmed cell death (Mollet et al., 2007) and physical changes that can cause mechanical damage (Acosta‐Martinez et al., 2010; Mardikar and Niranjan, 2000; McCoy et al., 2009, 2010). Therefore, a major challenge to successful commercializing of cell‐based therapies depends on the development of scalable manufacturing processes while maintaining potency, purity, and viability of the final live cell product (Carmen et al., 2012).

Most manufacturing processes include a clarification and concentration step of the cellular material derived from cell culture. Typically, a centrifugation or a filtration step is included to remove large aggregates, unwanted proteins, cell debris, and to concentrate the cellular suspension (Pattasseril et al., 2013). Cell‐based therapies to date use batch dead‐end centrifugation for cell concentration. However, centrifugation has proven to be a difficult step to automate while maintaining sterility (Mason and Hoare, 2006) as it requires the use of biological safety cabinets for the transfer stages. Therefore, if these therapies are to be commercialized at an industrial scale, batch centrifugation can signify a problem to achieve the required cell numbers for the manufacture of whole cell therapies (Lapinskas, 2010). Other alternatives such as continuous counterflow centrifugation and tangential flow filtration may be more amenable to large‐scale, contained and automated operations (Pattasseril et al., 2013). Counterflow centrifugation systems—like kSep® (KBI Biopharma) and Elutra cell separation system (Terumo BCT)—can wash cells, clear residuals from the supernatant and buffer exchange, while keeping the cells in suspension. kSep® in particular can be fully automated and contained, uses a disposable flow path processing 0.1 to thousands of liters, while achieving high cell recoveries (>80%) and high cell viabilities (>90%). The capital investment needed, coupled with the cost of disposables, needs consideration in process development (Pattasseril et al., 2013). Tangential flow filtration offers an alternative that can be scaled, automated, fully contained and, generally, with a lower capital investment and disposables costs, amenable to process development (Pattasseril et al., 2013).

Process development starts with the preparation of highly characterized cells using bench‐scale technologies and the focus of scale‐up is to reproduce this quality when preparing the cells at scale (Carmen et al., 2012). The development of bench‐scale technologies which better mimic the full scale would help significantly with this translation. A series of ultra scale‐down (USD) tools have been created to mimic various large scale operations such as continuous centrifugation (Boychyn et al., 2001; Hutchinson et al., 2006) and depth filtration (Jackson et al., 2006; Lau et al., 2013). USD devices allow the investigation of several manufacturing hydrodynamic environments (McCoy et al., 2009, 2010; Zoro et al., 2009) and geometries (Acosta‐Martinez et al., 2010) with little material, significantly increasing the throughput of the experimental phase in a cost‐effective and time‐efficient manner.

In this study, a modified version of the membrane separation USD device designed by Ma et al. (2010) was used to develop a methodology to investigate the effect of membrane processing on recovery of cells for therapy. The novelty lies in the use of mass balances across the membrane for the non‐invasive analysis of cell rupture as recorded by release of cytosolic lactate dehydrogenase (LDH) to give a rate of cell damage. The exclusion of trypan blue dye and the release of cytosolic LDH enzyme was used to characterize the final extent of cell damage in terms of loss of cell wall integrity (noted as loss of viability in this paper).

## Materials and Methods

### Cell Preparation

A human neonatal foreskin fibroblast cell line (HCA2 cells, provided by Prof David Kipling, University of Cardiff, UK) was cultured from frozen stock (passage 11) to 70–80% confluency (T175 flasks, Greiner Bio‐One, Frickenhausen, Germany) in Dulbecco's Modified Eagles Medium (DMEM, Invitrogen, Paisley, UK), supplemented with 10% (v/v) foetal calf serum (PAA Laboratories, Linz, Austria) and 2 mM of glutamine (Invitrogen). The supplemented medium will be referred as complete growth medium (hCGM). The cells were taken to a maximum of passage 25 over 60 days. For cell harvesting, the spent medium was removed; the cells were rinsed once with Dulbecco's Phosphate Buffered Saline (DPBS) (Sigma–Aldrich, Ayrshire, UK) and enzymatically detached using 5 mL of TrypLE™ Select (Invitrogen) for 5 min at 37°C. To quench the enzyme, an equal volume of growth media was introduced into the flask and the cells were centrifuged (Thermo, Strasbourg, France) for 3 min at 500 g and room temperature (21 ± 1°C). The cells were subsequently resuspended in ∼10 mL of growth medium to yield a suspension of ∼2 × 10^6^ cells mL^−1^and used within 5 min for membrane separation studies.

### Membrane Separation Studies

**Figure 1:**
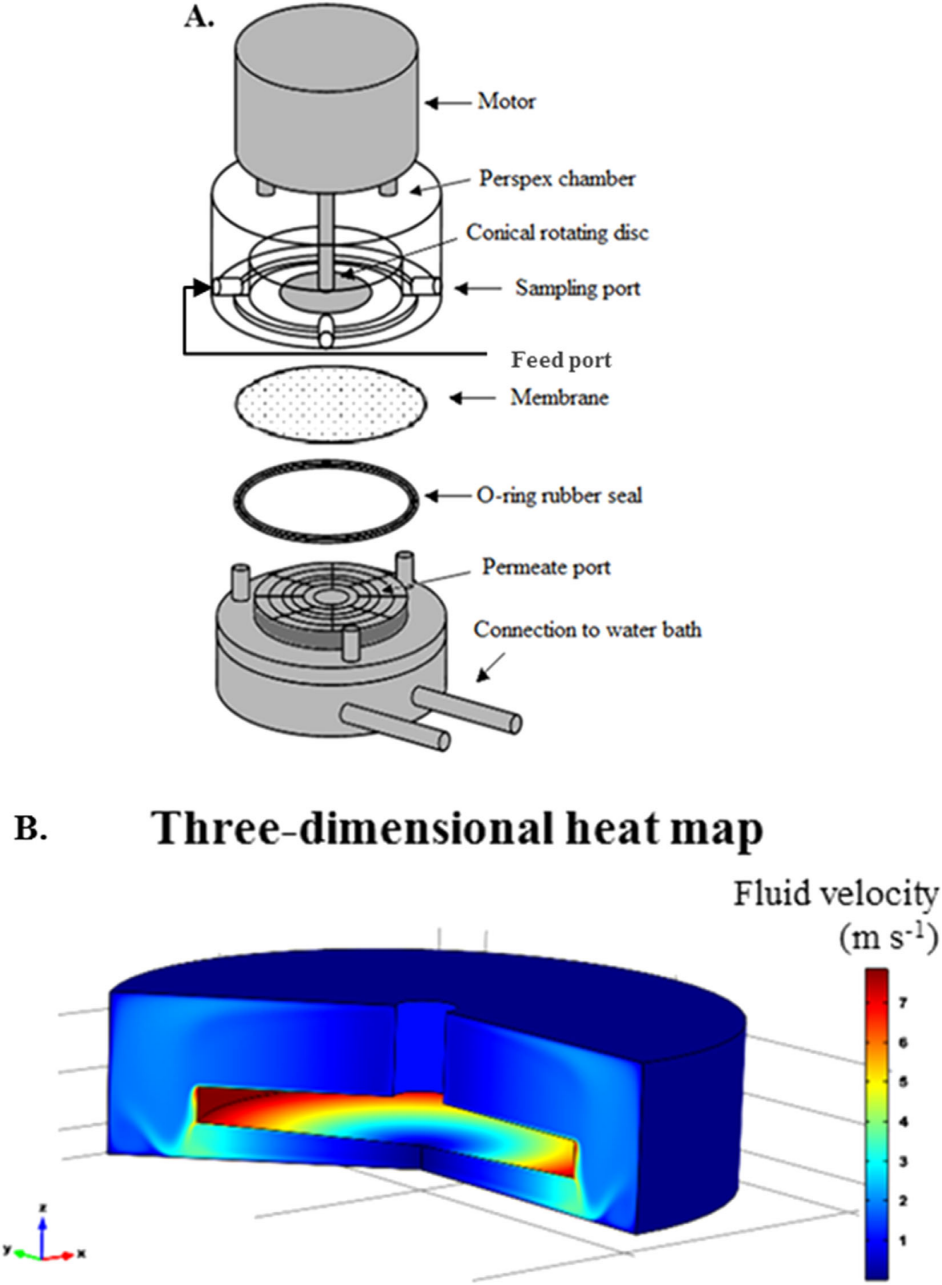
(A) An expanded view of the ultra scale‐down membrane filtration device (in this study the port located opposite that for sampling is used as the feed port), (B) computational fluid dynamic (CFD) simulation displaying the fluid velocity experienced at various locations of the USD membrane device at 10,000 rpm. Simulation assumes viscosity of 1 mPa s.

The membrane separation device used (see Fig. 1A) is similar to that described by Ma et al. (2010) and was built by the UCL Rapid Design and Fabrication Facility. It consisted of a centrally mounted conical disc, 15 mm diameter with a 4° conical cross‐section, with an accompanying shaft assembly fabricated from stainless steel. It is housed in a sealed Perspex chamber of internal diameter of 21 mm and a fixed total volume of 1.7 mL (Fig. 1A). A 25 mm diameter polyvinylidene fluoride Durapore® membrane (Millipore, Hertfordshire, UK) with 0.65 µm pore size, was mounted between the bottom of the shear cell and an O‐ring seal of 25 mm outer diameter that sits on the permeate port (Fig. 1A). Due to the width of the O‐ring, the effective area of the membrane is 3.64 cm^2^. A high speed motor with a feedback loop (Outrunner Motor, 920Kv Park 400, Champaign, Illinois) was used to give disc rotational speeds of 6,000 ± 50 and 10,000 ± 100 rpm. The chamber is fitted with a pressure sensor (RS Components Ltd, Northants, UK) connected to a multifunction data acquisition device (National Instruments Corporation Ltd, Berkshire, UK).

Computational fluid dynamics (CFD) software (Comsol Multipysics 3.4, Comsol, Hatfield, UK) was used to analyze the mean and maximum shear forces in the USD membrane separation chamber using methods described by Ma et al. (2010) for the establishment of mesh size and convergence. Figure 1B shows in a three‐dimensional heat map how the magnitude of the fluid velocity experienced by the material present in the retentate chamber varies with spatial location (assuming a density of water of 0.99 g cm^−3^ at 21 ± 1°C). It is evident that therefore the highest shear experienced is at the edges of the rotating disc (red areas which denote highest fluid velocity in Fig. 1B). The areas on top and below the rotating disc located away from the edges, exhibit a more even distribution of shear rates experienced by the fluid. For turbulent flow, the shear rate (γ) can be translated to energy dissipation rate (*ϵ* ≈ *P_W_*/*V*), which will be the parameter used in this paper, using the following relationship (Harrison et al., 2015):

(1) $$\gamma ={\left(\frac{P_{W}/V}{\rho \upsilon }\right)}^{1/2}$$

where *P_W_* is the power (*W*), ρ is the density of the fluid (assumed to be 1 × 10^3^ kg m^−3^) and υ is the kinematic viscosity of the fluid (assumed to be 1 × 10^−6^ m^2^ s^−1^). Local energy dissipation rates can be ∼10 times higher than the average rate and these probably determine cell damage, the focus of this paper. At 6,000 rpm disc speed, the average over the membrane surface and local (maximum at disc tip) energy dissipation rates are 0.2 and 1.9 W mL^−1^, respectively, (average and maximum shear rates of 14,000 and 44,000 s^−1^). At 10,000 rpm disc speed, local, and maximum energy dissipation rates equate to 1.4 and 13.5 W mL^−1^ (average and maximum shear rates of 37,000 and 116,000 s^−1^). Energy dissipation rate values quoted hereafter will be maximum energy dissipation rates (*ϵ*_max_). The shear rates used here are considerably higher than reported in literature, 3,000 s^−1^ (Cunha et al., 2015) and 4,000 s^−1^ (Rowley et al., 2012). This is to ensure that cell damage is recorded with a view to identifying conditions where no damage might occur, that is, to verify observations at full scale.

Cells were loaded using a disposable syringe, ensuring no air remaining in the chamber. The device was sealed and the cellular material was then sheared for 60 min at room temperature (21 ± 1°C). A 100 mL disposable syringe driven by a syringe pump (PHD 4400, Harvard Apparatus Ltd, Kent, UK) was used to feed growth media at a rate of 0.5 mL min^−1^ (equivalent flux rate of 82 LMH). The permeate was collected at 5 min intervals for 60 min. The chamber contents were collected at the end of the operation. A non‐sheared control was held in an Eppendorf tube concurrently, at the same temperature, for the duration of the experiment.

### Cell Suspension Analysis

Membrane integrity and concentration measurements were carried out, in quadruplicate, using the trypan blue exclusion method carried out in an automated,time‐controlled, haemocytometer (Vi‐Cell XR™, Beckman Coulter, Fullerton, CA). The device takes fifty images per sample and cell counting and analysis is carried out by using video imaging through a quartz flow cell. Membrane integrity (or percentage viability) is a measure of the proportion of viable cells to total cells as recorded by trypan blue exclusion.

### Cell Damage

LDH is a stable cytosolic enzyme that is released upon cell lysis. LDH was measured using a colorimetric assay (CytoTox‐96® Non‐Radioactive Cytotoxicity Assay, Promega UK, Southampton, UK) that quantitatively measures LDH released in the supernatant by using a 30 min coupled enzymatic assay which converts a tetrazolium salt (INT) into red formazan product.

The sample collected for LDH analysis was aliquoted into eight wells of a 96‐well round‐bottom plate (Nalgene Nunc International, New York), each containing 10,000–20,000 cells to obtain a reliable signal. A total of 10 µL of lysis solution (9% (v/v) Triton® X‐100) was added to four wells (technical repeats) for each sample, in order to measure the “total LDH release” (“LDH_TOT_” i.e., maximum release which includes extracellular or soluble LDH released to the medium as well as LDH from intact cells in the sample). A total of 10 µL of growth medium was added to the remaining four wells, to correct for volume. These wells were used to measure the “extracellular LDH release” (“LDH_EXT_” i.e., release from non‐viable and already lysed cells). “Intracellular LDH” (“LDH_INT_” i.e., LDH contained in viable cells with intact membranes) was calculated by the difference between the total and extracellular measurements.

The samples analyzed were: (i) the feed at time 0 (F, pre‐processing); (ii) the retentate at 60 min (PP, post‐processing); and (iii) the non‐sheared control (C) also measured at time 60 min.

### Mass Balances

**Figure 2:**
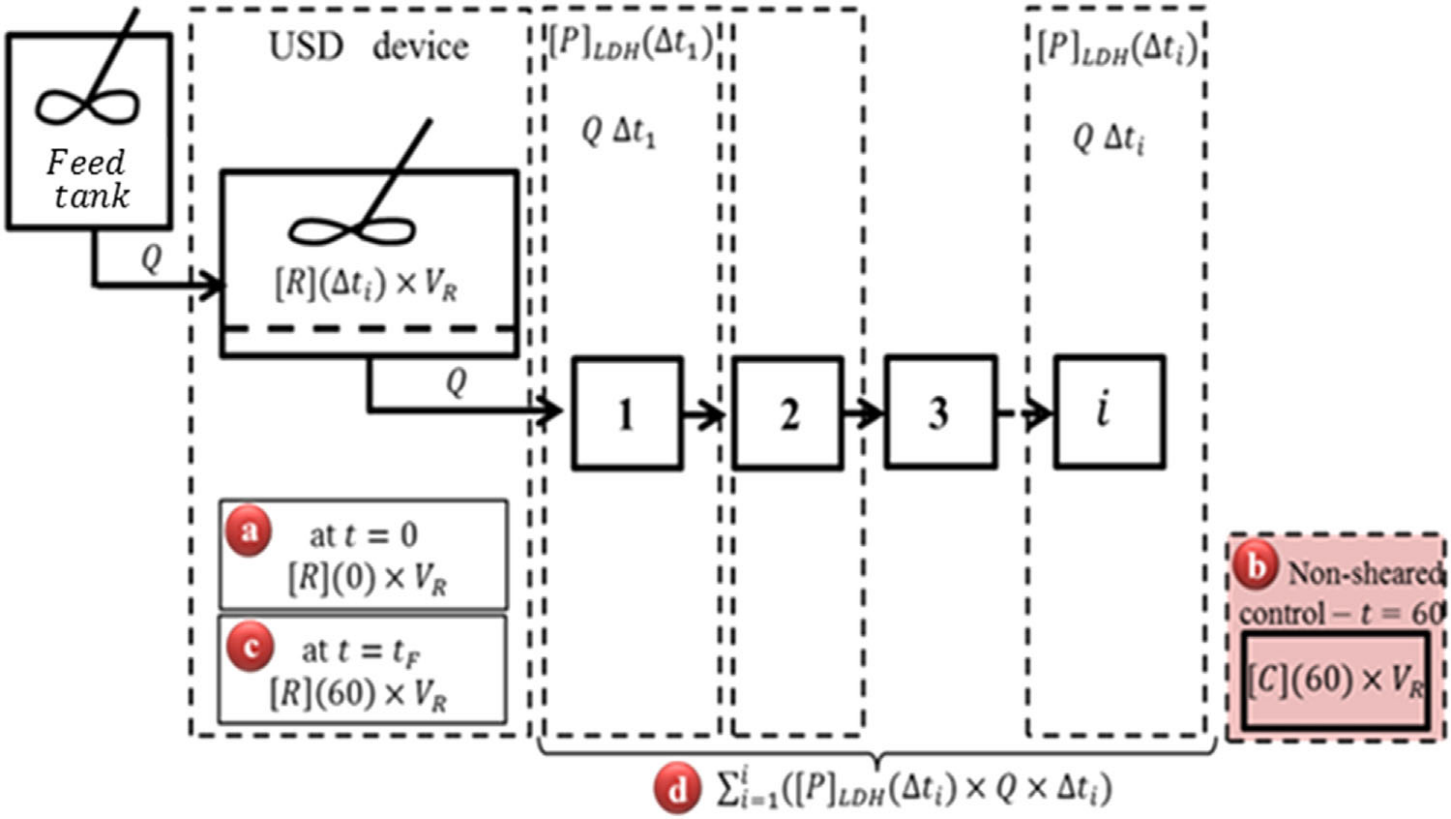
Block diagram for the experimental set‐up used to derive the amount of soluble LDH present during each time interval (Δ*t_n_*). The terms shown in (a) to (d), define the amount of LDH at various time points and locations in the experimental set‐up. (a) Initial soluble LDH, μU; (b) soluble LDH for non‐sheared control, μU; (c) soluble LDH remaining in retentate at end of study, μU; and (d) total LDH released and transmitted through membrane, μU.

Figure 2 shows the block diagram for the experimental set‐up used to derive the amount of soluble LDH present during each time interval (Δ*t_i_*). From left to right, a feed (growth media), *F*, is supplied to the USD membrane separation device at a constant flow rate, *Q*, by a syringe pump flushing growth media at a constant flux of 82 LMH for 60 min. The flux of choice and time of operation are in accordance with recent findings for larger scale (up to 2 L) TFF platform used by Cunha et al. (2015). The authors have shown for human mesenchymal stem cells (hMSCs), high cell recovery (>80%) and high percentage viability (>90%) post‐processing when controlling the permeate flux at 60, 120, and 250 LMH (time of operation of 92, 44, and 22 min, respectively).

**Figure 3:**
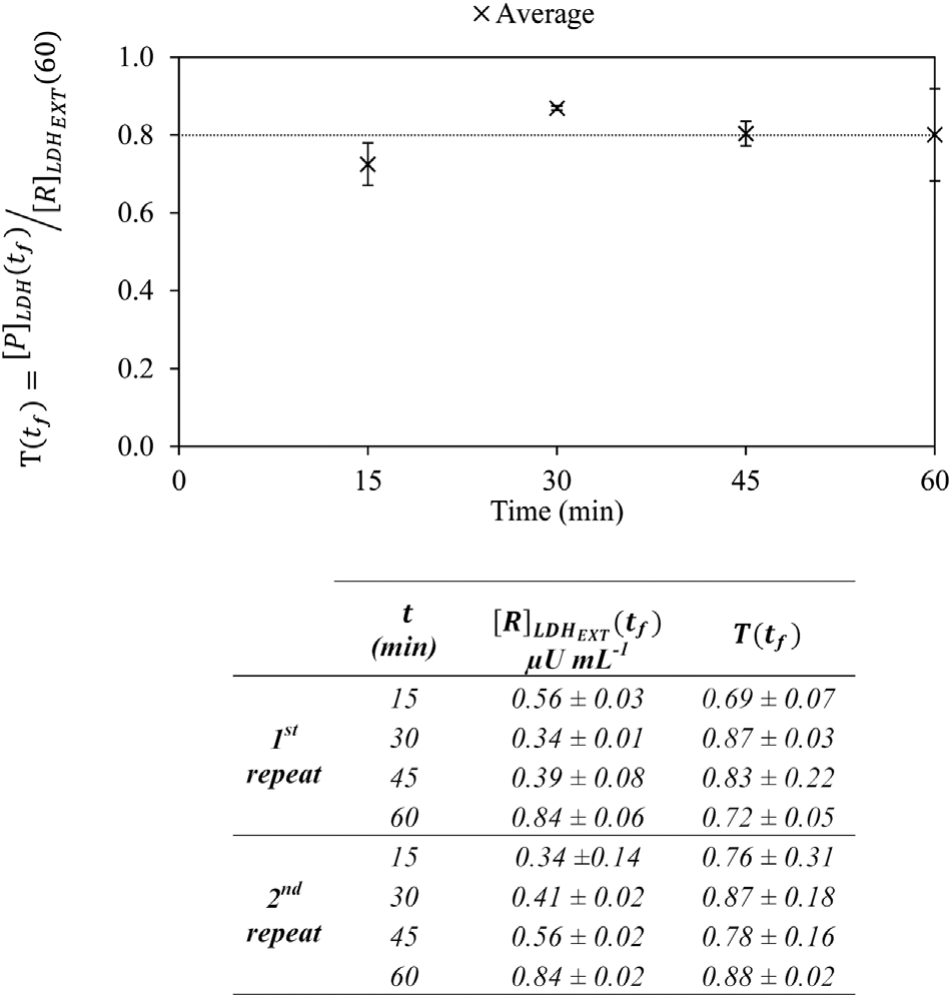
Effect of processing time on the measured transmission of LDH. The plot shows the average of two repeats carried out under the same operating conditions with two separately prepared cell suspensions (*Q* = 0.5 mL min^−1^, *V_R_* = 1.7 mL, 10,000 rpm, ${\left[R\right]}_{{TB}_{\text{TOT}}}$(0) ≈ 2.0 × 10^6^ cells mL^−1^). Dashed line shows the mean of *T*(*t_n_*) values from 15 to 60 min. The table below shows the raw data for each of the four separate experiments and both repeats.

For this study, the permeate stream, P, exiting the USD membrane separation device (labeled “Block USD device”) is pooled over 5‐min intervals, where “i” denotes the interval number, *i* = 1–12. The average concentration of extracellular LDH present in the retentate for any given time interval, Δ*t_i_*, is estimated using:

(2) $${\left[R\right]}_{{\text{LDH}}_{\text{EXT}}}\left(\Delta t_{i}\right)=\frac{{\left[P\right]}_{\text{LDH}}\left(\Delta t_{i}\right)}{T\left(t\right)}$$

where [*P*]_LDH_(Δ*t_i_*) is the measured concentration of LDH present in the permeate collected over interval Δ*t_i_*, subscript “LDH_EXT_” refers to extracellular component and *T*(*t*) is the LDH transmission at time *t*. Even though a 0.65 μm membrane is used, transmission values of <100% for LDH might be expected due the high concentrations of proteins present which will interact with the membrane. The transmission value, *T*(*t*), for 6,000 rpm was measured every 15 min intervals for 60 min as shown in Figure 3. An average transmission coefficient of 0.80 was recorded for *T*(*t_f_*) values ranging from 15 to 60 min with no significant trend noted with varying time. It was, therefore, concluded, that for both 6,000 and 10,000 rpm, a constant value of transmission may be assumed throughout the run to correct for the measured average concentration of LDH in the permeate over any particular time interval, and that value can be measured at the end of the run, *t* = *t_f_*:

(3) $$T\left(t_{f}\right)=\frac{{\left[P\right]}_{\text{LDH}}\left(t_{f}\right)}{{\left[R\right]}_{{\text{LDH}}_{\underline{EXT}}}\left(t_{f}\right)}$$

where *t_f_* is the final minute of processing, [*P*]_LDH_(*t_f_*) is the concentration of extracellular LDH present in the permeate collected over the period *t_f_* − 1 min to *t_f_* and [*R*]_LDHEXT_(*t_f_*) is the measured concentration of extracellular LDH in the retentate.

**Figure 4:**
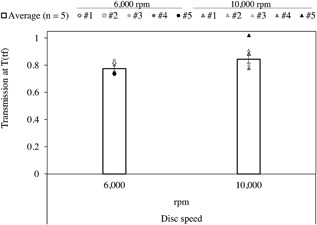
Transmission values calculated at time “*t_f_* ” for 6,000 and 10,000 rpm (*ϵ*_max_ ≈ 1.9 and 13.5 W mL^−1^, respectively), using measured concentration of soluble LDH in the retentate (“${\left[R\right]}_{{\text{LDH}}_{EXT}}$(60)”) and the permeate (“${\left[P\right]}_{LDH}\left(t_{f}\right)$”). Bars shown are mean values ± 1 s.e. (*j* = 5; *n* = 4) and individual runs are shown as discrete points (• 6,000 and △10,000 rpm). Average transmission calculated at 6,000 rpm is 0.78 ± 0.02 and 0.84 ± 0.03 at 10,000 rpm. All experiments were carried out at a concentration of ∼2 × 10^6^ total cells mL^−1^. Note run #5 at 10,000 rpm is an outlier and, therefore, not taken into account for average transmission value.

The experimental results for the LDH data for any of the runs, at any of the two given disc speeds, provides considerable information about the system. It informs on the concentrations of soluble LDH in the permeate and in the retentante over the final minute of operation (*t_f_*). These values are then used to calculate the transmission coefficient specific for that run. As shown in Figure 4, with the exception of one outlier at high disc speed, the reproducibility from one repeat to another is high, giving confidence in this measurement. It can, therefore, be concluded that average transmission values calculated at 6,000 and 10,000 rpm of 0.78 ± 0.02 and 0.84 ± 0.06, respectively, are representative of the individual repeats.

Other experimental data collected by LDH analysis also gives the following parameters over 5‐min intervals from 0 to 60 min: (i) the measured average concentration of soluble LDH collected in the permeate, [*P*]_LDH_; (ii) the calculated average amount of soluble LDH in the permeate based on the volume collected, *P*_LDH_; (iii) the calculated average concentration of extracellular LDH remaining in the retentate, ${\left[R\right]}_{{\text{LDH}}_{EXT}}$; (iv) the calculated average concentration of internal LDH in the retentate, ${\left[R\right]}_{{\text{LDH}}_{INT}}$; (v) the calculated proportion of intact cells remaining in the USD membrane separation device, *ω*.

Three main observations can be drawn from this data that applies to the individual runs: (i) the concentration of soluble LDH present in the permeate collected over the last minute of processing, [*P*]_LDH_(*t_f_*), is in agreement with the concentrations of LDH measured in the permeate stream during the last 10 min of operation; (ii) the highest concentrations of soluble LDH in the permeate, [*P*]_LDH_, were measured during the first 15 min of processing; (iii) the standard error for the concentration of extracellular LDH in the retentate post‐processing, ${\left[R\right]}_{{\text{LDH}}_{EXT}}$(60), gives confidence of the accuracy of this value as an estimate of the population mean. The above considerations are relevant to gain confidence on the value for the transmission coefficient, *T*(*t_f_*), because *T*(*t_f_*) is heavily dependent on both [*P*]_LDH_(*t_f_*) and ${\left[R\right]}_{{\text{LDH}}_{EXT}}$(60).

## Results and Discussion

By performing a mass balance of the LDH in the system, the proportion of intracellular LDH remaining in the USD membrane separation device versus time can be monitored. $R_{{\text{LDH}}_{INT}}$(0) is the calculated amount of intracellular LDH present in the USD membrane separation device at the start of the experiment (i.e., in the retentate at *t* = 0):

(4) $$R_{{\text{LDH}}_{\text{INT}}}\left(0\right)=R_{{\text{LDH}}_{\text{TOT}}}\left(0\right)-R_{{\text{LDH}}_{\text{EXT}}}\left(0\right)$$

where $R_{{\text{LDH}}_{TOT}}$(0) and $R_{{\text{LDH}}_{EXT}}$(0) are the measured total and extracellular amounts of LDH present in the USD membrane separation device at the start of the experiment, at *t* = 0 min.

The following relationships depend on the assumption that the total amount of LDH in the system (retentate and permeate) remains constant throughout the duration of the membrane separation study. For any particular interval, Δ*t_i_*, $R_{{\text{LDH}}_{INT}}$(Δ*t_i_*) is given by:

(5) $$R_{{LDH}_{\text{INT}}}\left(\Delta t_{i}\right)=R_{{LDH}_{\text{INT}}}\left(0\right)-\left(\sum_{i=1}^{i}P_{\text{LDH}}\left(\Delta t_{i}\right)+R_{{LDH}_{EXT}}\left(\Delta t_{i}\right)\right)$$

where *∑^i^*_*i *= 1_ *P*_LDH_(Δ*t_i_*) is the sum of the amounts of LDH recorded in the permeate stream collected from the start of the experiment to the end of interval Δ*t_i_* and $R_{{\text{LDH}}_{EXT}}$(Δ*t_i_*) is the calculated average amount of extracellular LDH present in the retentate during interval Δ*t_i_*.

The concentration $R_{{\text{LDH}}_{EXT}}$(Δ*t_i_*) is given by:

(6) $${\left[R\right]}_{{\text{LDH}}_{\text{EXT}}}\left(\Delta t_{i}\right)=\frac{{\left[P\right]}_{\text{LDH}}\left(\Delta t_{i}\right)}{T\left(t_{f}\right)}$$

where [*P*]_LDH_(Δ*t_i_*) is the concentration of extracellular LDH measured in the permeate collected over interval Δ*t_i_* and *T*(*t_f_*) is the transmission of LDH as measured from the retentate and permeate concentrations of LDH at the end of the experiment. Therefore, the average amount of extracellular LDH present in the retentate over interval Δ*t_i_* is given by:

(7) $${\left[R\right]}_{{\text{LDH}}_{\text{EXT}}}\left(\Delta t_{i}\right)=\frac{{\left[P\right]}_{\text{LDH}}\left(\Delta t_{i}\right)}{T\left(t_{f}\right)}\times V_{P}$$

where *V_P_* is the permeate volume over interval Δ*t_i_* (equal to *Q* × Δ*t_i_*). Hence from equations (5) and (7):

(8) $$R_{{\text{LDH}}_{\text{INT}}}\left(\Delta t_{i}\right)=R_{{\text{LDH}}_{\text{INT}}}\left(0\right)-\sum_{i=1}^{i}P_{\text{LDH}}\left(\Delta t_{i}\right)-\left(\frac{{\left[P\right]}_{\text{LDH}}\left(\Delta t_{i}\right)}{T\left(t_{f}\right)}\right)\times V_{P}$$

Using Values Measured Experimentally, Equation (8) Becomes:

(9) $$R_{{\text{LDH}}_{\text{INT}}}\left(\Delta t_{i}\right)=R_{{\text{LDH}}_{\text{INT}}}\left(0\right)\times V_{R}-\sum_{i=1}^{i}\left({\left[P\right]}_{\text{LDH}}\left(\Delta t_{i}\right)\times Q\times \Delta t_{i}\right)-\left(\frac{V_{P}\times {\left[P\right]}_{\text{LDH}}\left(\Delta t_{i}\right)}{T\left(t_{f}\right)}\right)$$

where *V_R_* is the volume of the retentate chamber. Hence:

(10) $$\omega \left(\Delta t_{i}\right)=\frac{{\left[R\right]}_{{\text{LDH}}_{\text{INT}}}\left(0\right)\times V_{R}-\sum_{i=1}^{i}\left({\left[P\right]}_{\text{LDH}}\left(\Delta t_{i}\right)\times Q\times \Delta t_{i}\right)-\left(\frac{V_{P}\times {\left[P\right]}_{\text{LDH}}\left(\Delta t_{i}\right)}{T\left(t_{f}\right)}\right)}{{\left[R\right]}_{{\text{LDH}}_{\text{INT}}}\left(0\right)\times V_{R}}$$

This parameter is calculated at 5‐min intervals using the LDH readings from the permeate. Therefore, to simplify equation (10), the proportion of intracellular LDH (that of intact cells) remaining in the USD membrane separation device, *ω*, versus time can be monitored and is given by:

(11) $$\omega \left(\Delta t_{i}\right)=\frac{R_{{\text{LDH}}_{\text{INT}}}\left(\Delta t_{i}\right)}{R_{{\text{LDH}}_{\text{INT}}}\left(0\right)}$$

**Figure 5:**
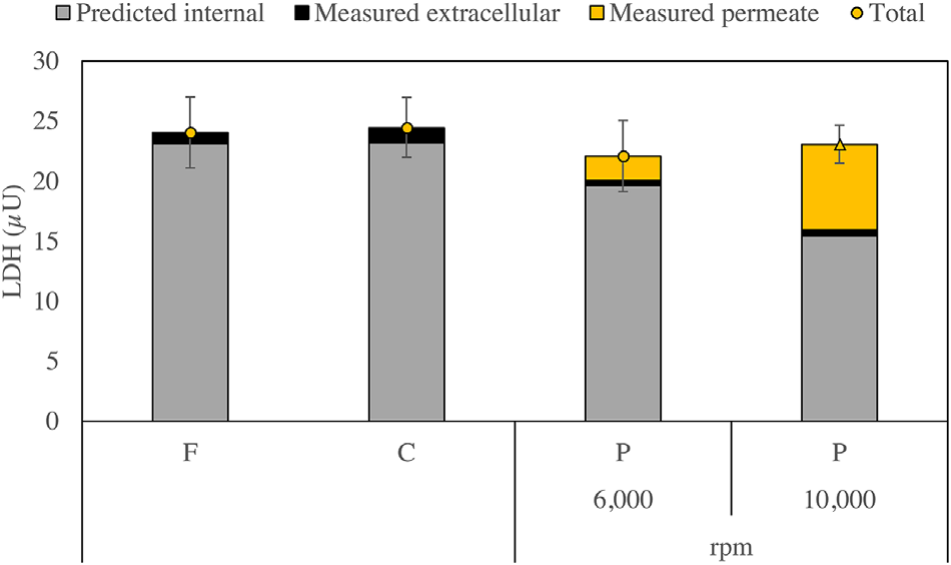
Amount of LDH measured and predicted for the feed (“*F*”), control (“*C*”), and post‐processing (“*P*”) samples at 6,000 and 10,000 rpm disc speeds (*ϵ*_max_ ≈ 1.9 and 13.5 W mL^−1^, respectively). The bars represent the cumulative LDH measured in the permeate stream (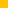), the measured soluble or extracellular LDH (▪) and the predicted internal LDH (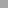). The individual points (• 6,000 rpm and ▴ 10,000 rpm) represent the total LDH (sum of permeate, extracellular and internal). All experiments were carried out at a concentration of ∼2 × 10^6^ total cells mL^−1^. The control is a non‐sheared sample held in a centrifuge tube concurrently, 21 ± 1ºC, for the duration of the experiment. High disc speed resulted in an increased amount of LDH measured in the permeate compared to low speed and, therefore, a decreased amount of predicted internal LDH. Data shown are mean values ± 1 s.e. (6,000 rpm *j* = 4 and *n* = 4; 10,000 rpm *j* = 5 and *n* = 4).

Figure 5 is a stacked bar chart which shows the measured amount of both total and extracellular LDH, as well as the calculated intracellular LDH for each of (a) the feed, F; (b) control, C; and (c) retentate post‐processing, PP, at 6,000 and 10,000 rpm. The cumulative amount of soluble LDH in the permeate stream, P_LDH_, is also shown on the post‐processing samples. Important information may be acquired from the interpretation of this figure such as: (i) there is no significant difference in the total LDH present in the feed and the non‐sheared control held for 60 min; (ii) there is good agreement in the amount of total LDH in the feed and that after processing. The first observation is of relevance to show that LDH was stable during the period of time measured and that the release of LDH is due to the effect of processing conditions and not an artifact of experimental procedure. These observations are in agreement with previous studies carried out by Berger and Tietz (1976) and Goldblum et al. (1990) and confirm that there is no loss of LDH activity by merely holding the sample without processing. Goldblum et al. (1990) measured LDH activity in insect cells every 30 min for 3 h showing no significant changes during this period of time. Moreover, Berger and Tietz (1976) reported LDH in serum to be stable for at least 3 days at room temperature.

Overall, from the LDH data in Figure 5 it is evident that processing at high disc speed for 60 min results in an increased amount of LDH measured in the permeate compared to low disc speed. The next section addresses the results by analysis of cell damage with time of operation for each individual run as well as the average of the five repeats at low and high disc speeds. It will also include an analysis on the trends observed with the trypan blue exclusion data.

### The Impact of Disc Speed (Maximum Energy Dissipation Rate) on Loss of Intact HCA2 Cells

Studies to evaluate the impact of disc speed on loss of intact cells were carried out for low (6,000 rpm) and high (10,000 rpm) disc speeds. These two speeds are equivalent to 1.9 and 13.5 W mL^−1^maximum energy dissipation rates, respectively. The major increase with greater speed is due to an enhanced axial flow circulation effects. The flow characteristics in the USD device will range from undeveloped laminar to turbulent flow while at full scale it would be expected that laminar conditions would prevail but with regions of repeated exposure to high stress in pumps and entry and exit locations. The USD device conditions chosen in this study reflect the higher stress regions which may occur (McCoy et al., 2010). Future work will require the match of USD conditions with those existing in different full scale operations. Four performance factors are used to characterize the effect on the cells of these disc speeds; (i) the transmission of LDH calculated at *t_f_*; (ii) the proportion of intact cells remaining as measured by LDH described in the previous section in Eq (11); (iii) the change in the population of viable cells; and (4) the change in percentage viability, both as measured by trypan blue exclusion.

**Figure 6:**
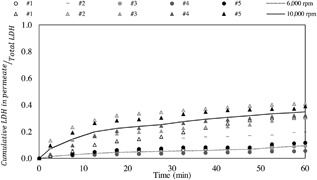
Cumulative LDH in the permeate stream over total LDH versus time at 6,000 and 10,000 rpm disc speeds (*ϵ*_max_ ≈ 1.9 and 13.5 W mL^−1^, respectively). All experiments were carried out at a concentration of ∼2 × 10^6^ total cells mL^−1^ except for run #2 at 6,000 rpm (−) which had a higher starting concentration of cells (∼4 × 10^6^ cells mL^−1^). Therefore, run #2 at 6,000 rpm is shown but not taken into account for best fit. The control is a non‐sheared sample held in a centrifuge tube concurrently, 21 ± 1ºC, for the duration of the experiment as defined by Figure 2 part (c) and typically showed no significant release of LDH over 60 min (data not shown).

The transmission values for each individual run that were previously shown in Figure 4, were used to calculate the amount of LDH in the permeate over time and is shown in Figure 6 as a proportion of the total amount of LDH measured. With the exception of run #2 at low disc speed, reproducibility from one repeat to another is high. Therefore, the average values (plotted as continuous lines instead of discrete points on Fig. 6) are deemed to be representative. Run #2 at low disc speed had an initial higher concentration (∼4 × 10^6^ cells mL^−1^) than all the other runs, approximately two times higher. This means the viscosity of the cell suspension would be ∼ three times higher (Zoro et al., 2009), potentially leading to higher energy dissipation rates experienced by the cells and the greater extent of cell damage measured. For the purpose of this paper, this run is shown but not considered when averaging the repeats.

**Figure 7:**
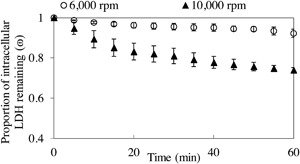
Membrane processing of feed (0 h cell ageing) HCA2 cells—effect of disc speed on cell damage as recorded by release of LDH (data averaged from Fig. 6). A non‐sheared control held in a centrifuge tube concurrently, 21 ± 1ºC, for the duration of the experiment was used to measure LDH pre and post‐processing. This figure shows the proportion of intracellular LDH remaining (*ɷ*, Eq (11)), for 6,000 rpm (○) and 10,000 rpm (▴) at a concentration of ∼2 × 10^6^ total cells mL^−1^ (*ϵ*_max_ ≈ 1.9 and 13.5 W mL^−1^, respectively). High disc speed resulted in a ∼30% reduction of viable cells as measured by release of LDH. Data shown are mean values ± 1 s.e. (6,000 rpm *j* = 4 and *n* = 4; 10,000 rpm *j* = 5 and *n* = 4).

Figure 7 shows the average values from Figure 6 of the four runs at low disc speed and five runs at high speed, carried out to analyze the impact of disc speed on cell damage as measured by LDH release. Overall, observing these results in Figure 7, the proportion of intracellular LDH remaining in the USD membrane separation device throughout operation decreased ∼8% at a low disc speed and ∼30% at a high disc speed. This finding suggests that increasing the speed of the disc, causes more damage to the cellular population being processed. Mass balances for both disc speeds (Fig. 5) reveal that the amount of total LDH in the non‐sheared controls held at 21 ± 1°C in a centrifuge tube concurrently for the duration of the experiment did not decrease. This leads to the conclusion that the changes seen at the two disc speeds can be fully attributed to processing conditions.

Moreover, when looking at the plots for the individual repeats (Fig. 7), the data shows the same profile of decrease with time. A combination of first order expressions could be used to describe these profiles. For the first 15–20 min of processing, the rate of damage to intact cells is faster than the remaining 40–45 min of processing. This indicates a fairly evident weaker population of cells to start with, followed by a more robust remaining cell population. It may be that if processing was prolonged, the second population may continue to be progressively damaged until there are no cells left in the USD membrane separation device. Previous studies on cell damage carried out by Midler and Finn (1966), observed that the damage of protozoa cells in a uniform shear device showed a two phase behavior; the first phase being a rapid primary damage followed by a slow decline of viable cells. They concluded that cell damage may not only be related to magnitude of the hydrodynamic forces but also to the exposure time. From our study, this may be the case but it is not a straightforward and certain observation to test due to the number of complications that this implies. Prolonged processing time may cause cell damage by the introduction of other variables such as cell ageing while processing or change in the processing medium properties due to release of intracellular components.

**Figure 8:**
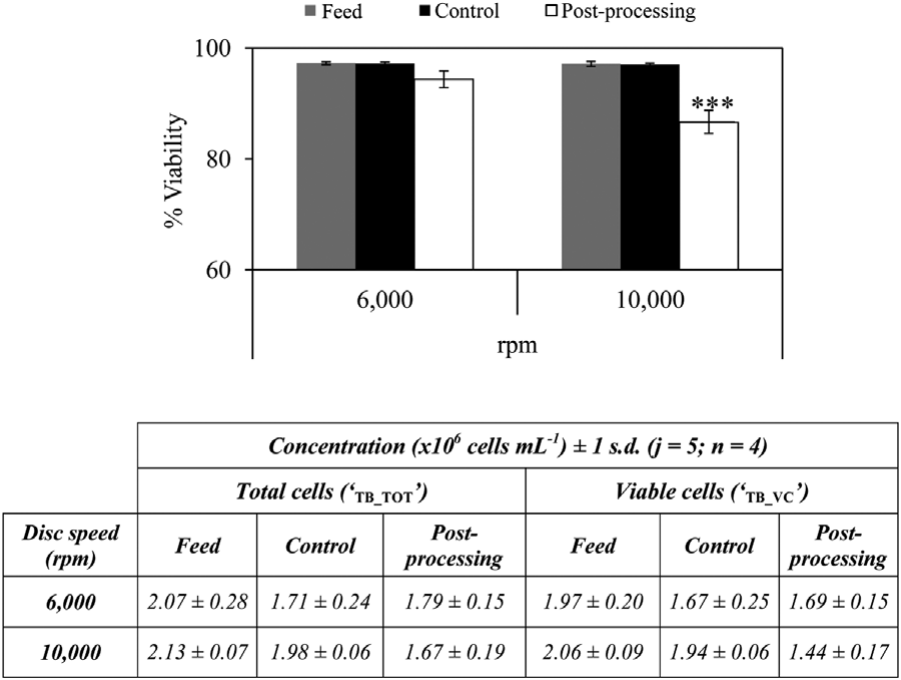
Membrane processing of feed (0 h cell ageing) HCA2 cells—effect of disc speed on percentage viability of the cellular suspension as recorded by trypan blue exclusion. A non‐sheared control held in a centrifuge tube concurrently, 21 ± 1ºC, for the duration of the experiment was used to measure trypan blue exclusion pre‐ and post‐processing for both 6,000 and 10,000 rpm (*ϵ*_max_ ≈ 1.9 and 13.5 W mL^−1^, respectively). Trypan blue exclusion indicates that processing at high disc speed, resulted in a ∼10% drop in percentage viability post‐membrane processing. Low drop was observed in percentage viability at a low disc speed. Significant changes between non‐sheared control and post‐processing (*P < 0.5, **P < 0.01, ***P < 0.001). Table below shows mean of raw data determined by trypan blue exclusion for concentration of total and viable cells. Processing at high disc speed, resulted in a ∼25% reduction of viable cells. Data shown are mean values ± 1 s.d. (6,000 rpm *j* = 4 and *n* = 4; 10,000 rpm *j* = 5 and *n* = 4).

Figure 8 was used to analyze the impact of disc speed on cell damage as measured by trypan blue exclusion. It shows the percentage viability for the feed, control and post‐processing samples and both disc speeds. After harvesting the cells and prior to processing, high percentage viabilities are observed (∼97.3% as shown by the feed samples). These high percentages are maintained when the cells are held at 21 ± 1°C in a centrifuge tube concurrently for the duration of the experiment (∼97.1% as shown by the control samples). Further analysis of Figure 8 shows that high disc speed resulted in a significant ∼10% (*P* = 0.001) drop in percentage viability post‐processing compared to no drop in percentage viability at a low disc speed.

The legend for Figure 8 shows a table with the concentration of total and viable cells for the same three samples. The average concentrations of viable cells in the control samples are 1.97 ± 0.20 and 2.06 ± 0.09 × 10^6^ cells mL^−1^ for low and high disc speeds respectively. At high disc speed there is a significant ∼25% (*P* = 0.003) decrease in the population of viable cells, compared to no decrease at a low disc speed.

Overall, the comparison of the performance factors of the feed samples to the controls, showed that holding the cells at 21 ± 1°C in a centrifuge tube concurrently for the duration of the experiment did not lead to spontaneous release of LDH or drop in percentage viability. Moreover, with respect to cell damage, LDH release showed similar trends and amounts of damage for both low and high disc speed as trypan blue exclusion method.

### The Impact of Disc Speed (*ϵ*_max_) on Morphology of HCA2 Cells in Suspension

It has been widely reported that changes in physical appearance and morphology can occur after the cells are exposed to stresses (Al‐Rubeai et al., 1995a; Kretzmer and Schügerl, 1991) and can serve as indicators of the state of cell health (Agashi et al., 2009; Kretzmer and Schügerl, 1991). Within this section, the morphology and physical appearance of cells post‐processing was investigated. Morphological analysis was carried out on the cellular suspension immediately post‐processing and 2 h post‐processing.

**Figure 9:**
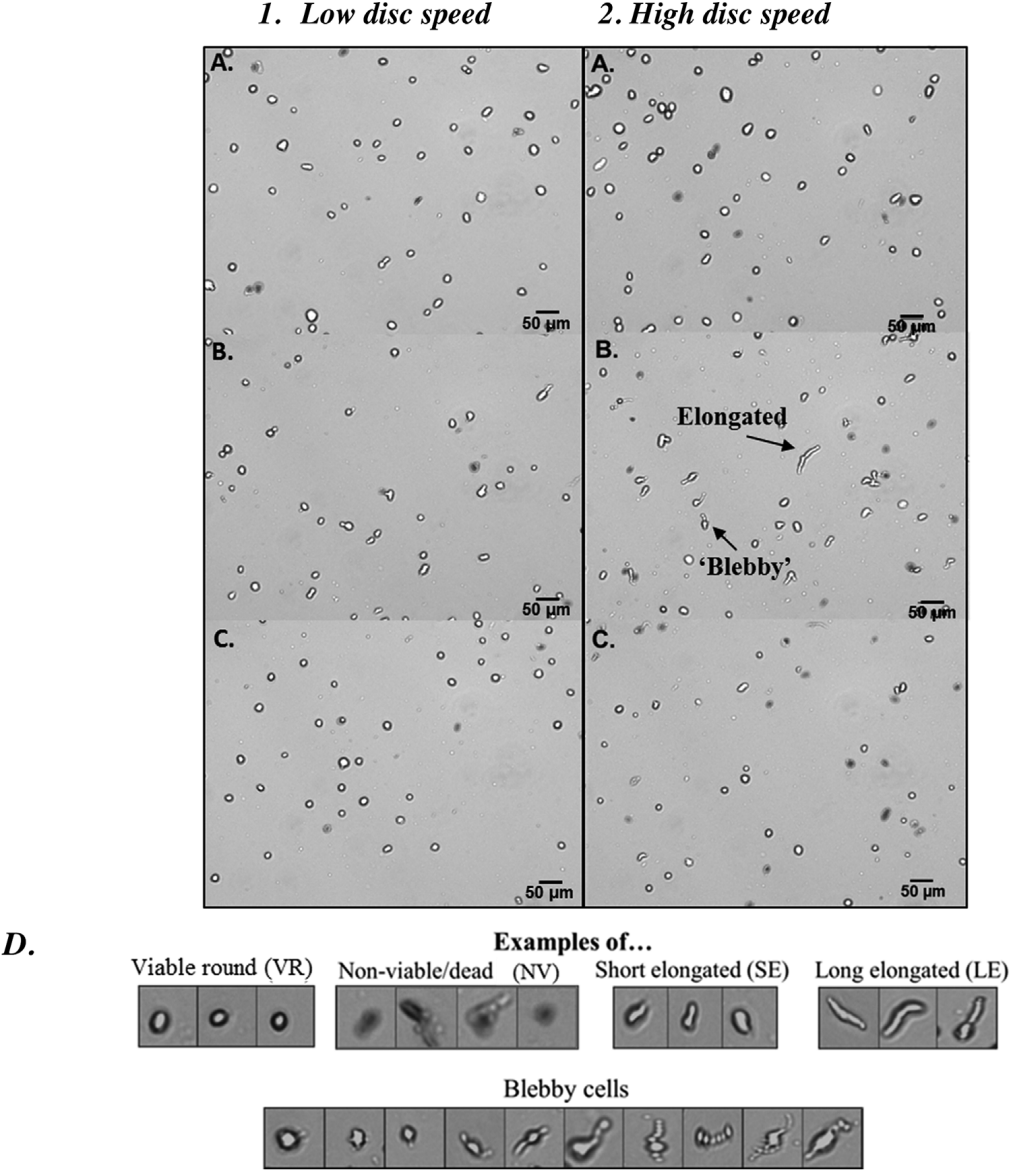
Images of (A) feed; (B) post‐processing, and (C) 2 h hold post‐processing at low (1) and high (2) disc speeds (6,000 and 10,000 rpm, respectively). Figure (D) shows examples of HCA2 images from the cell image library created to facilitate the identification of cell phenotype in a given image. The cell library was constructed from images selected for each designated category. Each category was assigned parameters (such as aspect ratio and intensity) and these were then used as rules for identification purposes for the Matlab script.

Figure 9 shows some examples of the type of images obtained from the automated haemocytometer. For both low and high disc speeds (Fig. 9(1) and (2), respectively) three time points are shown; feed samples (labeled A), immediately post‐processing (labeled B) and 2 h hold post‐processing (labeled C), all at 21 ± 1°C.

From Figures 9(1A) and (2A), feed samples for both low and high disc speeds, it can be seen that most cells are round viable cells with sharply defined outer edges or clearly non‐viable as stained positive for trypan blue exclusion. However, the appearance of elongated and “blebby” cells is observed immediately post‐processing (Fig. 9(1B) and 9(2B)). Because “blebbing” can be a characteristic of cell death, preliminary analysis of measurement of programmed cell death (data not shown) using Caspase3—a key molecule in the apoptotic mechanism and induction of apoptosis (Darzynkiewicz and Pozarowski, 2007; Smolewski et al., 2002)—was carried out and indicated increased activity post‐processing. “Blebs” are membrane bound protrusions from the plasma membrane caused by weakness in the actin cytoskeleton which helps maintain cellular structure (Kumar et al., 2007). Therefore, the potential of these being “blebs” would need to be studied further using f‐actin stain. Literature reveals that “blebbing” is a dynamic process and can resolve itself once the actin cytoskeleton is restored (Kumar et al., 2007) and in fact, images C, after 2 h hold post‐processing, reveal that a large portion of the “blebs” have disappeared and there are lower numbers of elongated cells. This trend seems to be more evident at high disc speed (Fig. 9(2)) than at low disc speed (Fig. 9(1)).

**Figure 10:**
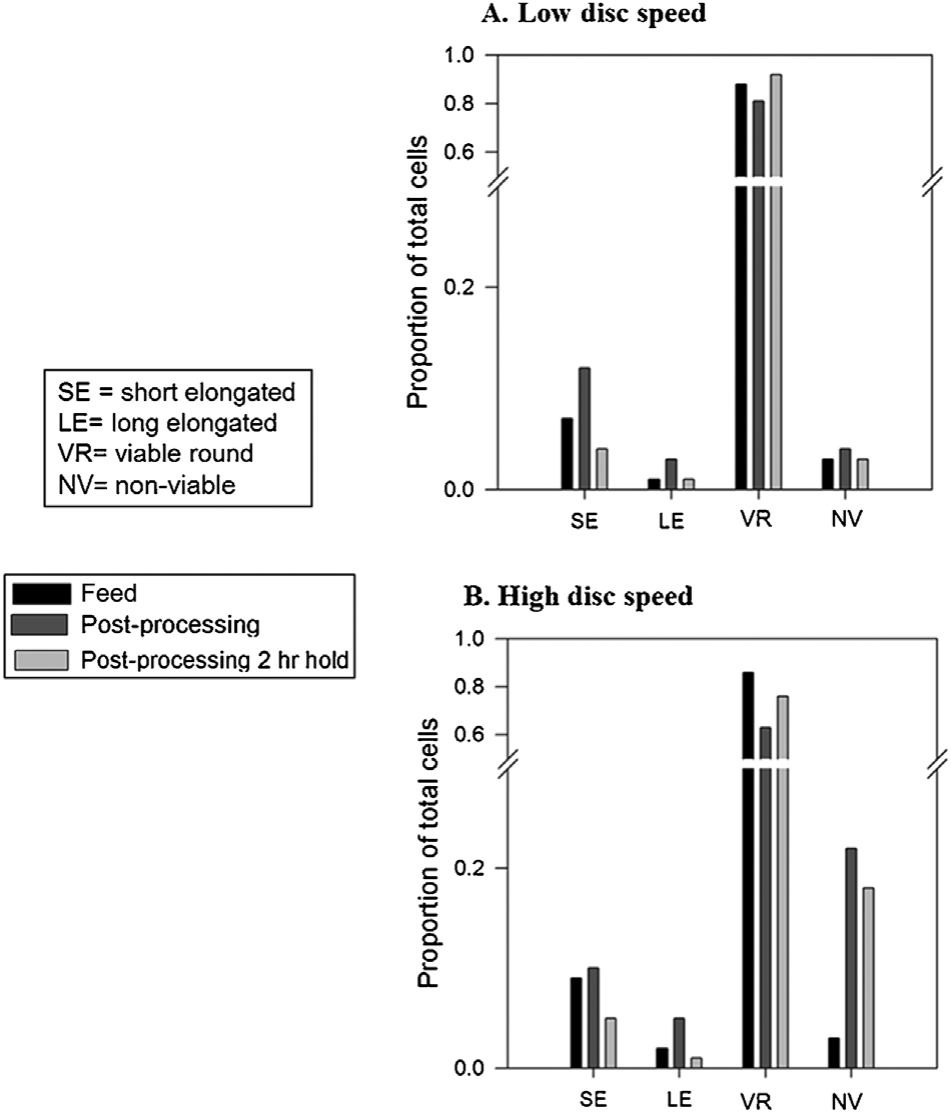
Effect of disc speed (A) 6,000 rpm and (B) 10,000 rpm on cell morphology after shear and after shear plus hold, quantified by software for image processing developed using Matlab Image Processing Toolbox (MathWorks, Cambridge, UK) in the Biochemical Engineering Department (UCL). The cell categories correspond to libraries created for the Matlab script (Fig. 9D). The proportion of each cell type or population was compared to the total cells in the sample for feed (▪), immediately post‐processing (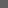), and 2 h hold post‐processing (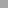).

These morphological characteristics identified were used as building blocks to define the different cell types to develop a script for image processing using MATLAB Image Processing Toolbox (MathWorks, Cambridge, UK). Figure 9D shows some examples for each of the cell types. The cells were classified into short elongated (SE), long elongated (LE), viable round (VR), non‐viable (NV), and debris (DEBRIS). For example, parameters such as aspect ratio were used to classify cells into round, short or long elongated cells; intensity was used to identify dead and alive and area was used to identify debris from cells. Originally, a degree of “blebbing” for each cell type was going to be included as an added characteristic to each cell population. However, the implementation and incorporation of this parameter proved to be unreliable. The images from the automated haemocytometer were analyzed using the Matlab script and the proportions for each of the cell populations at the three given time points (feed, immediately post‐processing and 2 h hold post‐processing) are shown in Figure 10A and B (low and high disc speeds respectively).

From Figure 10B, high disc speed, it can be seen that there is an increase in the proportion of dead cells and a reduction in the proportion of viable round cells immediately post‐processing that remained the same after 2 h hold. For both low and high disc speeds (Fig. 10A and B, respectively) there is an increase in the proportion of both short and long elongated cells immediately post‐processing that appears to return to feed sample levels after the 2 h hold. The apparent trend previously appreciated in Figure 9 is in agreement with the quantitative data in Figure 10.

A temporary change of morphology is appreciated by the appearance of elongated and blebby cells post‐processing, most of which appear to resolve themselves after 2 h of hold. This is not to say that categorizing the state of cell health on physical appearance alone would be sufficient, nonetheless it may serve as a fast and inexpensive way of preliminary assessment of cell health.

## Conclusion

The focus of this study was a clinically relevant human fibroblast (mesenchymal) cell line. Tests with cell lines such as human mesenchymal stem cells (hMSCs) would be key to establish a broader relationship for the prediction of full scale processing. The greater heterogeneity compared with the mesenchymal cells used here, might lead to different relations between loss of cell membrane integrity and processing conditions. Furthermore, a wider range of cell characterizations assays (e.g., immunocytochemistry, flow cytometry, cell proliferation) would be needed, especially when conditions are established of no loss of cell membrane integrity. Studies at both higher (applicable to concentration operations) and lower (applicable for example to the initial harvest of hMSCs) concentrations, will probably lead to different sensitivities to the imposed shear conditions.

A methodology to assess cell damage as measured by LDH release into the permeate of the USD membrane separation device was developed, tested and supported by trypan blue exclusion results. Investigation of the impact of disc speed on cell damage confirmed that the decrease in the proportion of intracellular LDH remaining in the USD membrane separation device throughout processing was higher at high disc speed than at low disc (∼30% compared to ∼8%). Trypan blue exclusion method also revealed a higher decrease in the population of viable cells at high disc speed than at low disc speed (∼25% compared to no drop). Moreover, high disc speed resulted in a significant drop of ∼10% in viability post‐processing compared to no drop at a low disc speed as measured by trypan blue exclusion. It appears that LDH release may be a more sensitive indicator of earlier damage to the cell membrane than trypan blue exclusion, observation which has been previously documented by Lappalainen et al. (1994). Lastly, morphological analysis of cells in suspension revealed the appearance of elongated and “blebby” cells immediately post‐processing, most of which disappear after a 2 h hold; trend which was more apparent when operating at high disc speed than at low disc speed.

The authors would like to thank the support from; the BBSRC support for FM and the Regenerative Medicines Bioprocessing Group at UCL Biochemical Engineering (Prof. C. Mason, Dr L. Ruban, Dr N. Jaccard (for Matlab script for cell morphology) and Dr S. Gerontas.

## Nomenclature

A: soluble LDH present (μU)
C: control
F: feed
i: interval number
j: number of runs
n: number of measurements
P: permeate
P_w_: power (W)
PP: post‐processing
Q: flow rate (mL min^−1^)
R: retentate
t: time (min)
T: transmission
V: volume (mL)

### Symbols

[]: concentration (cells mL^−1^ or μU mL^−1^)
Δ: change
∑: sum
ϵ_max_: maximum energy dissipation rate, P_W_/V (W mL^−1^)
ɷ: proportion of intracellular LDH remaining
γ: shear rate (s^−1^)
ρ: density (kg m^−3^)
υ: kinematic viscosity (m^2^ s^−1^)
μ: viscosity (mPa s)

### Subscripts and Superscripts

EXT: external
f: final
INT: internal
LDH: lactate dehydrogenase
TB: trypan blue
TOT: total
